# Reactive Palladium–Ligand Complexes for ^11^C-Carbonylation at Ambient Pressure: A Breakthrough in Carbon-11 Chemistry

**DOI:** 10.3390/ph16070955

**Published:** 2023-07-03

**Authors:** Kenneth Dahl, Anton Lindberg, Neil Vasdev, Magnus Schou

**Affiliations:** 1 PET Science Centre, Precision Medicine and Biosamples, Oncology R&D, AstraZeneca, Karolinska Institutet, SE-17176 Stockholm, Sweden; magnus.schoul@astrazeneca.com; 2 Department of Clinical Neuroscience, Centre for Psychiatry Research, Karolinska Institutet and Stockholm County Council, SE-17176 Stockholm, Sweden; 3 Azrieli Centre for Neuro-Radiochemistry, Brain Health Imaging Centre, Centre for Addiction and Mental Health, 250 College St., Toronto, ON M5T1R8, Canada; anton.lindberg@camh.ca; 4 Department of Psychiatry, University of Toronto, 250 College St., Toronto, ON M5T1R8, Canada

**Keywords:** carbonylation, radiochemistry, carbon-11, Xantphos, radiopharmaceuticals

## Abstract

The Pd–Xantphos-mediated ^11^C-carbonylation protocol (also known as the “Xantphos- method”), due to its simplistic and convenient nature, has facilitated researchers in meeting a longstanding need for preparing ^11^C-carbonyl-labeled radiopharmaceuticals at ambient pressure for positron emission tomography (PET) imaging and drug discovery. This development could be viewed as a breakthrough in carbon-11 chemistry, as evidenced by the rapid global adoption of the method by the pharmaceutical industry and academic laboratories worldwide. The method has been fully automated for the good manufacturing practice (GMP)-compliant production of novel radiopharmaceuticals for human use, and it has been adapted for “in-loop” reactions and microwave technology; an impressive number of ^11^C-labeled compounds (>100) have been synthesized. Given the simplicity and efficiency of the method, as well as the abundance of carbonyl groups in bioactive drug molecules, we expect that this methodology will be even more widely adopted in future PET radiopharmaceutical research and drug development.

## 1. Introduction

Positron emission tomography (PET) is a highly sensitive molecular imaging modality that plays a vital role in medical diagnosis, most predominantly within oncology, cardiology, and neurology [1,2,3,4]. PET imaging has also become a key technology to support drug development [5]. The use of radiopharmaceuticals, which are labeled with short-lived radionuclides, e.g., carbon-11 (^11^C, t_1/2_ = 20.4 min) and fluorine-18 (^18^F, t_1/2_ = 109.7 min), in combination with PET and other imaging modalities (e.g., MRI or CT) allows for the quantitative assessment of tissue access, target engagement, and the pharmacological effect of small molecule- and peptide-based drug candidates. The successful use of PET in clinical research and diagnosis heavily relies upon the availability of suitable radiochemical methods to prepare appropriate molecular probes that are labeled with positron-emitting radionuclides [6].

**Figure 1:**
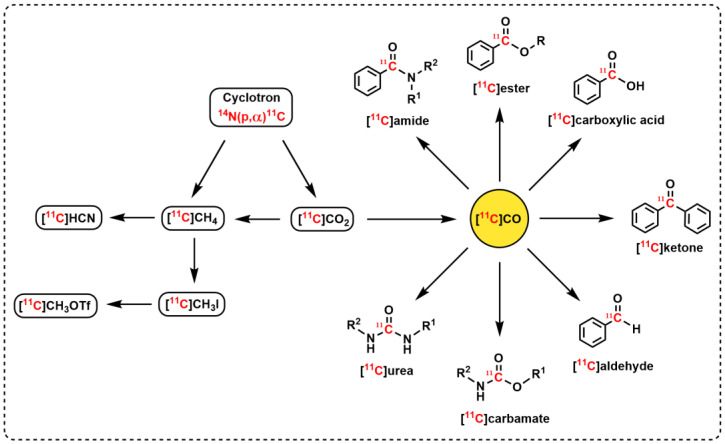
Nuclear formation of carbon-11 and its transformation to commonly used ^11^C precursors, with the versatility of the [^11^C]CO building block for the labeling of diverse functional groups in PET radiochemistry.

Carbon-11 is one of the most useful radionuclides for PET radiochemistry as its naturally occurring isotope, carbon-12, is present in all organic molecules. Labeling with carbon-11 can thus be achieved without altering the physicochemical or pharmacological properties of a compound. Carbon-11 is generally produced with a cyclotron using a high-energy proton bombardment of nitrogen gas according to the ^14^N(p, α)^11^C nuclear reaction [7,8,9,10]. The two major ^11^C-labeled primary precursors, [^11^C]carbon dioxide ([^11^C]CO_2_) and [^11^C]methane ([^11^C]CH_4_), are formed, respectively, when either small amounts of oxygen (0.1–2%) or hydrogen (5–10%) are present in the target. These simple precursors are sometimes directly used for radiopharmaceutical synthesis; however, most frequently, [^11^C]CO_2_ and [^11^C]CH_4_ are converted into secondary ^11^C precursors via various rapid and efficient on-line procedures (Figure 1), most noteworthily [^11^C]methyl iodide, [^11^C]methyl triflate, [^11^C]hydrogen cyanide, and [^11^C]carbon monoxide ([^11^C]CO) [6]. The secondary precursor [^11^C]CO has been increasingly recognized as an important ^11^C-synthon for PET radiopharmaceutical research and production [11,12,13,14,15]. In this review, we highlight the development of a novel ^11^C-carbonylation technology, referred to as the “Xantphos-method”, that has allowed laboratories to meet a longstanding, unmet need for preparing ^11^C-carbonyl-labeled radiopharmaceuticals at ambient pressure for PET imaging [16]. This article will cover the chemical and technical aspects of the method, any further developments or improvements since its discovery, automation for clinical translation, and its use within PET radiopharmaceutical development.

## 2. ^11^C-Carbonylation

**Figure 2:**
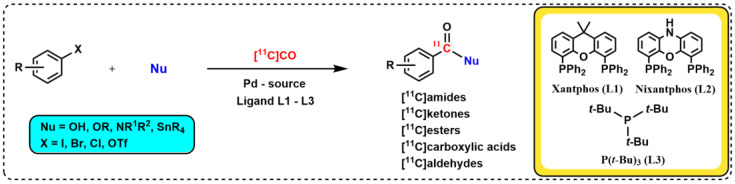
General description for the three-component carbonylation to form ^11^C-carbonyl containing products using activated Pd–ligand complexes.

Carbonyl groups are one of the most widespread functional groups in bioactive drug molecules, and transition metal-catalyzed coupling reactions in the presence of carbon monoxide represent an efficient way to introduce a carbonyl group into various organic compounds [17]. [^11^C]CO has many attractive features as a building block for PET radiochemistry, which include its facile production and high versatility in transition metal-mediated carbonylation reactions. Various ^11^C-labeled functional groups can be prepared from [^11^C]CO, including [^11^C]amides, [^11^C]esters, [^11^C]carboxylic acids, and [^11^C]ketones (Figure 1) [12,13,14,15]. In addition, [^11^C]CO synthesis is technically straightforward and involves a one-step gas phase reduction of the cyclotron produced, [^11^C]CO_2_, over heated zinc (400 °C) or molybdenum (850 °C). The molybdenum reduction method is generally preferred due to its greater reproducibility over time [15]. However, despite this great potential of [^11^C]CO as a ^11^C-building block for PET radiochemistry, its widespread use has long been hampered by the lack of a simple method for its introduction. The most explored methodology to date is the high-pressure autoclave method [18,19]; however, due to its technically sophisticated setup, the focus has recently been directed to different ways of performing [^11^C]CO-carbonylation reactions at ambient pressure. To date, there are four successful methods for this reaction at ambient pressure reported in the literature: (i) the chemical complexation of [^11^C]CO with diborane (BH_3_-[^11^C]CO) [20] or copper scorpionates (Cu-Sc-[^11^C]CO) [21]; (ii) the application of xenon gas as a carrier to allow the quantitative transfer of [^11^C]CO into a sealed reaction vessel [22]; (iii) the oxidant-assisted methoxy ^11^C-carbonylation of aryl boronates [23]; and (iv) carbonylation using highly reactive Pd–ligand complexes (Figure 2, e.g., Pd–Xantphos, also known as “Xantphos-method”) [16]. The latter method is the focus of this review.

### 2.1. Discovery and Development of the Pd–Xantphos Protocol for ^11^C-Carbonylation Radiochemistry at Ambient Pressure

**Figure 3:**
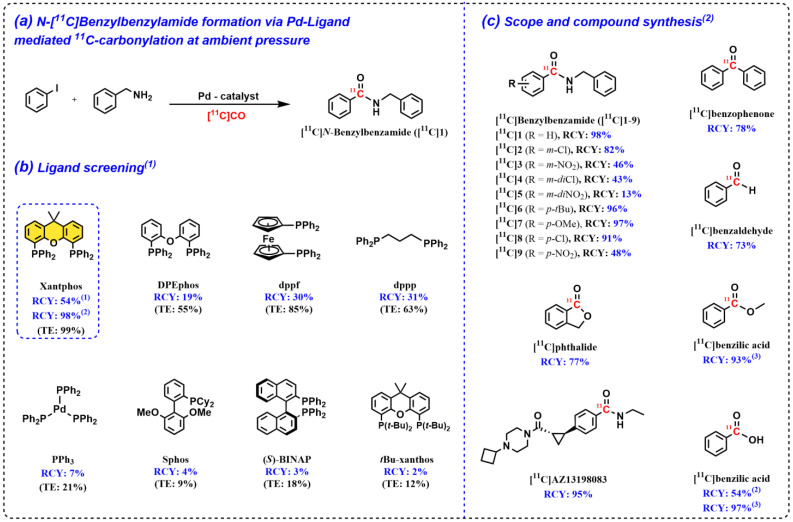
Pd–Xanthos-mediated carbonylation to form an ^11^C-carbonyl-containing product at ambient pressure. ^(1)^ Ligand screening was performed with Pd(OAc)_2_ as the Pd catalyst. ^(2)^ Yields were obtained using Pd_2_[π-cinnamyl]Cl_2_. ^(3)^ Experiments were performed using microwave heating.

Xantphos, a bidentate ligand developed by van Leeuwen et al. in 1995 for the hydroformylation reaction, has been extensively used for homogeneous Pd catalyst reactions since its discovery [24]. The wide bite angle (110°) and flexibility range (97–133°) characteristic are believed to induce a dynamic coordination environment that could be of importance for catalyst activity and stability in transition metal-catalyzed processes [25]. Interestingly, the carbonylative coupling reaction appears to be a process for which bidentate ligands, such as Xantphos, have a unique ability to produce a highly active catalytic system, as has been reported in several studies [26,27,28,29]. In particular, the study reported by Buchwald et al. [26,27] highlighted the importance of Xantphos-based ligands for carbonylation that was carried out at low carbon monoxide pressure (1 atm). Inspired by this pioneering work, in early 2013, we published a groundbreaking study where it was discovered that the Pd–Xantphos complex provided a markedly better [^11^C]CO trapping efficiency (TE) and reaction selectivity compared with other examined phosphine-based ligands (Figure 3a,b) [16]. The reaction was carried out at ambient pressure in a conventional 4 mL disposable glass reaction vessel, which followed a single pass of pre-concentrated [^11^C]CO through a solution containing a Pd catalyst, a supporting ligand, a halide substrate, an appropriate nucleophile dissolved tetrahydrofuran (THF), and most importantly, the absence of any additional trapping agents or high-pressure components. The developed method only relies on the use of the highly active Pd–ligand complex, which is derived from Pd_2_(π-cinnamyl)Cl_2_-Xantphos; this enables the high trapping efficiency (>99%) of the sub-microgram amounts of [^11^C]CO in the reaction solution, followed by an almost instant carbonylative formation of the desired ^11^C-carbonyl-labeled product. The method was used to effectively label a range of *N*-[^11^C]benzylbenzamides (Figure 3c, [^11^C]1–9) and was applied to the ^11^C-labeling of a ketone, carboxylic acid, lactone and an aldehyde (Figure 3c) [16]. As a testament to the utility of this method, a candidate radioligand for the histamine type-3 receptor, [^11^C]AZ13198083, was also prepared (Figure 3c). Immediately following this initial study, we investigated the effect of microwave heating on ^11^C-carbonylation chemistry [30]. Improved radiochemical yields (RCYs) were obtained for the ^11^C-aminocarbonylation of electron-deficient aryl halides compared with thermal heating, and the approach even allowed for the use of an aryl chloride as substrate. The scope of this reaction was further extended to the labeling of an [^11^C]carboxylic acid and two [^11^C]esters (Figure 3c). Overall, this novel ^11^C-carbonylation technology represents a simple and straightforward path to the [^11^C]CO labeling of drug molecules and radiopharmaceuticals.

### 2.2. Pd–NiXantphos: An Alternative Pd-Ligand Complex for ^11^C-Carbonylation

**Figure 4:**
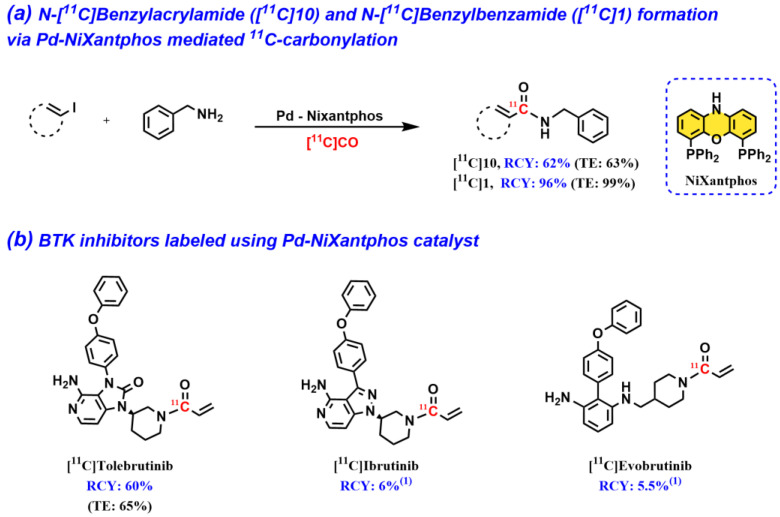
Pd–NiXantphos-mediated carbonylation to form ^11^C-carbonyl containing products. ^(1)^ Non-decay corrected and isolated radiochemical yields relative to [^11^C]CO_2_ at the start of synthesis.

Subsequently, we extended the scope of the current methodology to include another Pd–ligand complex, namely, Pd–NiXantphos [31]. We developed Pd–NiXantphos as a superior catalyst for [^11^C]acryl amide formation and applied it in the radiosynthesis of [^11^C]tolebrutinib, an isotopologue of the Bruton’s tyrosine kinase (BTK) inhibitor from Sanofi’s pipeline (Figure 4a,b). Inspired by this work, the *N*-[^11^C]acrylamide moiety of [^11^C]tolebrutinib was labeled via Pd–NiXantphos-mediated carbonylation with [^11^C]CO, iodoethylene being used as a substrate, and through the corresponding secondary amine precursor. The synthesis was automated using a commercial carbon-11 synthesis platform (TracerMaker™, Scansys Laboratorieteknik). In addition to labeling of *N*-acrylamides, the protocol was also applied in the synthesis of an aromatic amide (Figure 4a). The Pd–NiXantphos method has since been used for the radiosynthesis of [^11^C]ibrutinib [32] and [^11^C]evobrutinib [33] (Figure 4b). [^11^C]evobrutinib was successfully synthesized in the presence of an amino base to aid in the solubility of the amine-containing precursor. It is important to note, however, that *N*-[^11^C]acrylamide has been previously synthesized from [^11^C]CO using Pd(PPh_3_)_4_ as a catalyst [34,35,36]. Prior to this work, ^11^C-labeled *N*-acrylamides were most commonly synthesized via the intermediate formation of [^11^C]acrylic acid or the corresponding [^11^C]acryloyl chloride through the carboxylation of Grignard reagents with [^11^C]CO_2_ [37]. However, unlike Grignard reagents, which require great care and the rigorous exclusion of atmospheric moisture and CO_2_ during storage and manipulation, the Pd-catalyzed reactions reviewed herein are mild, chemoselective, and possess diverse functional group tolerance.

### 2.3. Isolated Aryl–Pd–Ligand Complexes for ^11^C-Carbonylation Radiochemistry

**Figure 5:**
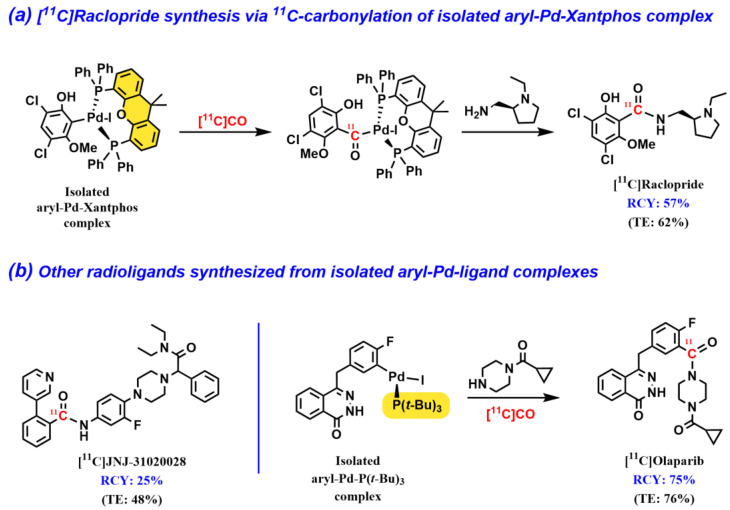
^11^C-Carbonylative synthesis of [^11^C]raclopride, [^11^C]JNJ-31020028, and [^11^C]olaparib using isolated aryl–Pd–ligand complexes.

Andersen et al. presented an interesting improvement of the current ^11^C-carbonylation protocol, where pre-isolated aryl–Pd–ligand complexes were developed as reagents to be utilized as precursors for the following carbonylative ^11^C-labeling reaction [38]. As these isolated complexes have already undergone the oxidative addition step prior to use in ^11^C-labeling, high RCYs were obtained for the synthesis of three structurally diverse and pharmaceutically relevant compounds (Figure 5). Once again, the Pd–Xantphos complexes appeared to be among the most reactive precursors, as apparent for the successfully preparation of the well-known dopamine D_2_ receptor PET radioligand [^11^C]raclopride (traditionally prepared by standard ^11^C-methylation) and neuropeptide Y_2_ receptor antagonist [^11^C]JNJ-31020028 (Figure 5a,b). Furthermore, another Pd–ligand complex was identified as a valuable catalyst for ^11^C-carbonylation at ambient pressure, namely Pd–P(t-Bu)_3_ (Figure 5b). In addition to providing high TE and carbonylation yields, this catalyst was found to be instrumental in preventing arene scrambling from phosphine ligands bearing phenyl groups (e.g., Xantphos and triphenylphosphine) when electron-deficient aryls were used as substrates. This was exemplified in the radiosynthesis of the poly (ADP-ribose) polymerase (PARP) inhibitor [^11^C]olaparib (Figure 5b). More recently, the same group presented a similar approach to selectively label peptides at the N-terminal or at the internal lysine position using [^11^C]CO [39]. The described method relies on the use of an isolated methyl–Pd–Xantphos complex to facilitate the production of native N-^11^C-acetylated peptides. The protocol was applied in the synthesis of three bioactive peptides.

### 2.4. “In-loop” ^11^C-Carbonylation Reaction

The captive solvent (“in-loop”) methodologies have been widely adapted for the routine production of ^11^C-radiopharmaceuticals because of their simplicity, high RCYs, speed, and ease of automation. Based on the previous success of this technology for ^11^C-radiochemistry, we recently reported the development of a simplified “in-loop” ^11^C-carbonylation method for labeling PET radiopharmaceuticals with [^11^C]CO [40]. The coupling reagents were simply loaded into the HPLC loop of the radiochemistry system prior to the start of synthesis. Without using any additional solid support, [^11^C]CO was directly trapped in the loop coated with the reaction solution, which was reacted at a room temperature (RT)-100 °C for 5 min and then injected onto a semi-preparative HPLC column for further purification. Notably, the Pd–Xantphos as well as the Pd–P(t-Bu)_3_-based systems were both shown to be compatible with this novel methodology. The “in-loop” process was used to label a number of carbonyl functional groups, including [^11^C]amides, [^11^C]esters, and [^11^C]carboxylic acids in moderate-to-excellent yields, and were applied in the synthesis of four drug-like molecules, namely [^11^C]olaparib, [^11^C]raclopride, [^11^C]FLB457, and [^11^C]AZ13198083. Recently, Scott, Shao, and co-workers developed an automated and good manufacturing practice (GMP)-compliant “in-loop” ^11^C-carbonylation procedure using a modified GE TracerLab^TM^ synthesis platform [41]. In this study, the standard Pd–Xantphos catalytic system was replaced with Pd–NiXantphos due to its greater solubility in THF to prevent clogging of the HPLC loop and injector. Using the optimized conditions for “in-loop” ^11^C-carbonylation, three clinically relevant BTK inhibitors, [^11^C]ibrutinib, [^11^C]tolebrutinib, and [^11^C]evobrutinib, were synthesized at good RCYs. Based on the known advantages associated with “in-loop” reactions, this method may become a valuable alternative methodology for the ^11^C-carbonylation of drugs and radioligands for PET imaging.

### 2.5. TracerMaker^TM^: A Fully Automated and GMP-Compliant Synthesis of [^11^C]CO-Labeled Radiopharmaceuticals

**Figure 6:**
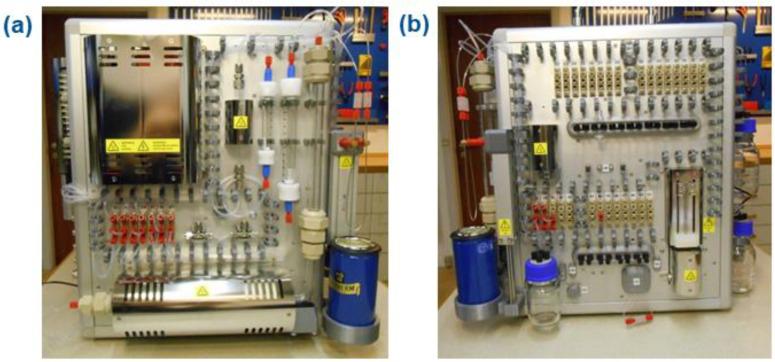
Images of the TracerMaker™ System. (**a**) Gas handling system: on-line preparation of [^11^C]CO, [^11^C]CO_2_ [^11^C]CH_3_I, and [^11^C]CH_3_OTf. (**b**) Liquid handling system: labeling reaction, purification, and formulation.

Several recent reviews have concluded that the lack of commercially available synthesis modules for ^11^C-carbonylations and GMP production is among the most important challenges to overcome for dissemination of [^11^C]CO chemistry to the wider community [14,15]. In an effort to bring wide access to this versatile ^11^C-synthon, we partnered with an experienced carbon-11 module developer, namely Dr. Peter Larsen (Scansys Laboratorieteknik, Copenhagen, Denmark), with the aim to develop the first commercially available and fully automated synthesis unit for ^11^C-carbonylations. The result of our collaboration was presented in 2020 [42]. The developed [^11^C]CO radiochemistry apparatus was designed to handle all parts of a radiopharmaceutical production, which include: (i) the initial on-line reduction of in-target produced [^11^C]CO_2_ to [^11^C]CO; (ii) transferring the concentrated [^11^C]CO into a single-use glass vessel (4 mL) for the radiolabeling reaction; and (iii) purification of the crude product solution and formulation following HPLC and solid phase extraction (SPE). The prototype apparatus was also applied to the production and clinical validation of the histamine type-3 receptor radioligand [^11^C]AZ13198083. The obtained product fulfilled quality control (QC) specifications. Following this work, Scansys Laboratorieteknik implemented the [^11^C]CO-specific components (e.g., column oven for the Mo-mediated reduction of [^11^C]CO_2_ and the [^11^C]CO trap) to their next generation carbon-11 platform, TracerMaker™, which is an all-in-one ^11^C-chemistry module that allows for the ^11^C-labeling of terminal methyl groups using standard ^11^C-methylation as well as the radiolabeling of the carbonyl moiety using either [^11^C]CO_2_ or [^11^C]CO (Figure 6). We have recently exemplified the utility of this versatile synthetic platform in the diverse radiochemistry presented in a series of publications [31,32,33,43,44]. In addition to this commercially available module, the well-established Synthra™ ^11^C-synthesis platform developed by Dr. Bruno Nebeling (Synthra GmbH, Hamburg, Germany) has recently implemented the synthesis of [^11^C]CO as an add-on feature for the advanced Synthra™ MeIplus Research module. Interestingly, this module also has the option to integrate a loop reactor with heating and cooling capabilities. This combination may be an ideal solution to expand commercial access to the novel “in-loop” ^11^C-carbonylation described in Section 2.4. 

## 3. Pd–Xantphos-Based Radiochemistry for the Synthesis of Bioactive Molecules and Their Applications in PET Imaging

**Figure 7:**
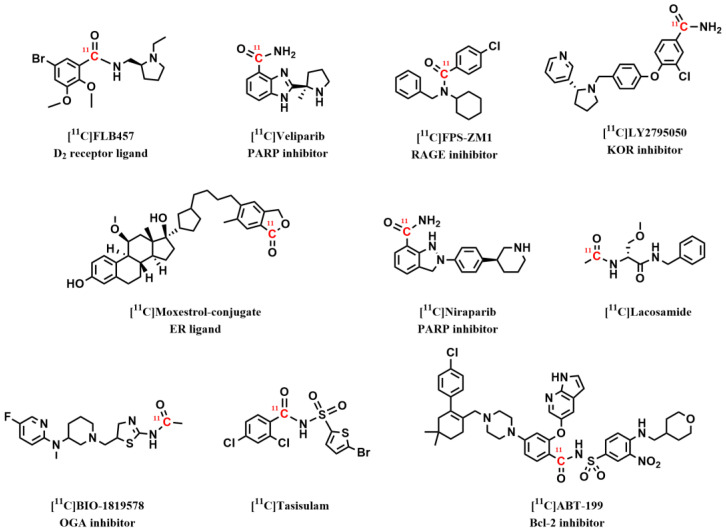
Drug-like compounds labeled using Pd–Xanthos-based ^11^C-carbonylation radiochemistry.

Since its discovery, the “Xanthos-method” has been fully automated for the GMP-compliant production of novel radiopharmaceuticals, adapted for “in-loop” reactions and microwave technology, and has most recently been translated for human use (data not yet published). An impressive number of labeled compounds (>100) have been synthesized by our laboratories and others. In fact, in a recent [^11^C]CO review by Eriksson et al., the authors concluded that a great majority of all published ^11^C-aminocarbonylation protocols in the past decade have been performed using Pd–Xantphos complexes [15]. Figure 7 illustrates the compounds that have been synthesized by Pd–Xanthos-based ^11^C-carbonylation radiochemistry, which are further discussed in Section 3.1 and 3.2.

### 3.1. Radiopharmaceuticals for PET Imaging in the Central Nervous System

Molecular imaging using PET radiopharmaceuticals can afford a sensitive and relatively non-invasive quantitation of biochemical processes within the central nervous system (CNS); so far, most of the radiopharmaceuticals developed via the Pd–Xantphos-mediated protocol has been within the CNS space. Our laboratory recently synthesized three candidate radioligands targeting the histamine type-3 receptor (H_3_R) [45], namely [^11^C]AZ13153556, [^11^C]AZD5213, and [^11^C]AZ13198083 (Figure 3). H_3_R is widely expressed in the CNS and plays an important role in regulating the release of various neurotransmitters. Radioligands were labeled in high yields (≥80%) using the Pd–Xantphos-mediated ^11^C-aminocarbonylation protocol. All three compounds showed high permeability into a non-human primate (NHP) brain and displayed regional distribution in accordance with known H_3_R brain expression. [^11^C]AZ13198083 had the most favorable in vivo kinetics, with high initial heterogenous uptake followed by a progressive washout from the NHP brain. Furthermore, pre-treatment and displacement studies using AZD5213 showed that [^11^C]AZ13198083 had high specific uptake and reversible binding. Collectively, this work demonstrates that [^11^C]AZ13198083 is a promising candidate for H_3_R imaging and is suitable for PET imaging in human subjects. 

The well-established dopamine D_2_ antagonist radioligand, [^11^C]raclopride (Figure 5), which is commonly produced via *O*-^11^C-methylation, has also been prepared using the Pd–Xantphos method (vide supra) [46]. The automated process, which was developed by Rahman et al., provided [*carbonyl*-^11^C]raclopride in a good overall yield and molar activity (RCY = 50 ± 5%, Am = 34 GBq/µmol). PET studies were conducted in monkeys to facilitate a direct comparison of the in vivo characteristics for [*carbonyl*-^11^C]raclopride and [*O*-*methyl*-^11^C]raclopride. Interestingly, both radioligands displayed similar results with respect to protein binding, radiometabolism, and quantitative outcome measures; therefore, the authors concluded that demethylation is not the prime route of radioligand metabolism. Moreover, the structurally similar D_2_ PET radioligand [^11^C]FLB457 (Figure 7) was recently ^11^C-labeled in the carbonyl position via an “in-loop” Pd–Xantphos protocol in good yield (RCY = 42%); however, no in vivo PET evaluation of this isotopologue was reported [40]. 

The receptor for advanced glycation endproducts (RAGE) is increasingly recognized as a viable target for the early detection of Alzheimer’s disease using PET. In 2019, Luzi et al. presented the synthesis and in vitro evaluation of a RAGE antagonist [^11^C]FPS-ZM1 that was labeled using [^11^C]CO (Figure 7), which possesses nanomolar affinity for RAGE (Ki = 25 nM). [^11^C]FPS-ZM1 was obtained in good and reproducible yields (RCY = 9.5%) and moderate molar activity (Am = 0.77 ± 0.13 GBq/µmol) [47]. A possible explanation for the rather low Am obtained for this radioligand could be the unconventional method used for the [^11^C]CO_2_ to [^11^C]CO conversion [48]. This in-solution method requires great care during reagent preparation to prevent contamination from atmospheric CO_2_. Nonetheless, the produced ligand was of sufficient quality for in vitro autoradiography evaluation. Despite its high affinity for RAGE, [^11^C]FPS-ZM1 displayed dense and non-displaceable binding to mouse brain tissues, and no significant difference was observed between the wild-type versus transgenic AD mouse model. The poor radioligand displacement was attributed to the high lipophilicity of [^11^C]FPS-ZM1. The findings outlined in this study may partly help to guide future PET radioligand efforts toward the development of second-generation RAGE PET imaging agents. 

The kappa opioid receptor (KOR) plays an important role in the regulation of brain functions and is involved in a variety of neurologic and psychiatric diseases. [^11^C]LY2795050 is a promising PET radioligand for the visualization of the KOR in the living human brain (Figure 7). This radioligand is currently prepared using a technically challenging and two-step metal-mediated ^11^C-cyanation protocol. However, most recently, Kaur et al. developed a simplified “in-loop” approach to synthesize [^11^C]LY2795050 in one step via a Pd–Xantphos-mediated ^11^C-carbonylation reaction [49]. The developed method provided [^11^C]LY2795050 in a near quantitative yield and is currently being validated for human use. 

O-linked-β-N-acetyl-glucosamine hydrolase (OGA) is an enzyme that regulates the production of the intracellular protein O-GlcNAc. O-GlcNAc formation is believed to reduce tau aggregation, which is a hallmark for Alzheimer’s disease. An industrial–academic partnership (Biogen, USA, and Karolinska Institutet, Sweden) recently labeled an OGA inhibitor as a potential PET radioligand [50]. The radioligand, which was named [^11^C]BIO-1819578, was successfully prepared by using a Pd–Xanthos-mediated ^11^C-carbonylation reaction performed at ambient pressure (Figure 7). PET studies in cynomolgus monkeys revealed that [^11^C]BIO-1819578 has high brain uptake (SUV = 7) as well as specific binding to the OGA enzyme. These results indicate that [^11^C]BIO-1819578 is a promising candidate for OGA imaging in human subjects. 

### 3.2. Radiopharmaceuticals for PET Imaging in Oncology

Another major area of interest for PET radiopharmaceuticals development is oncology. The relatively short half-life of carbon-11 has limited the development of the “Xantphos-method”-based small-molecule ^11^C-radiopharmaceuticals for peripheral oncology targets, as longer-lived radionuclides generally provide a better match to the pharmacokinetics of radiotracer binding. Nonetheless, noteworthy examples of ^11^C-carbonylated PET tracers are described below.

Poly (ADP-ribose) polymerase (PARP) is an enzyme involved in the DNA repair process and has been an interesting target for imaging tumors using PET. The synthesis of [^11^C]olaparib from [^11^C]CO using the “Xantphos-method” was first reported by Andersen et al. (Figure 5) [38]. The radiosynthesis has also been shown using the “in-loop” methodology [40]. [^11^C]olaparib has not been reported in PET imaging as of yet; however, its isotopologue [^18^F]olaparib has been shown to image PARP-expressing tumors in transgenic mice [51,52]. Furthermore, in a recent study, Ferrat et al. presented a convenient two-step method for the preparation of ^11^C-labeled primary benzamides, including two potent PARP inhibitors, [^11^C]niraparib and [^11^C]veliparib (Figure 7) [53]. The carbonylative protocol relied on the initial formation of [^11^C]aroyl dimethylaminopyridinium ([^11^C]aroyl-DMAP) salts as potent electrophiles to facilitate the acylation of an amine precursor compound. This current method paves the way for the future application of [^11^C]aroyl-DMAP salts in PET radiopharmaceutical development. 

Bruton’s tyrosine kinase (BTK) inhibitors are currently under exploration for diverse cancers and neurological disorders, and they have been a target of large interest for PET radioligand development (vide supra). The most known irreversible BTK inhibitors contain an acrylamide functionality (e.g., ibrutinib, tolebrutinib, and evobrutinib), as this moiety undergoes a Michael addition, forming covalent bonds to the BTK protein. In a number of studies, the *N*-acrylamide moiety has been targeted as a potential site for ^11^C-labeling with [^11^C]CO [31,32,33,41]. When this manuscript was being written, [^11^C]ibrutinib was the only BTK ligand prepared for in vivo PET studies [32]. Our preliminary PET–MRI studies with [^11^C]ibrutinib in experimental autoimmune encephalomyelitis (EAE) mice showed higher radioactivity accumulation (49%) in the spinal cord compared with sham mice. 

B-cell lymphoma 2 (Bcl-2) is an anti-apoptotic protein that is a promising therapeutic target in hematologic malignancies. Venetoclax (ABT-199) was developed as a selective Bcl-2 inhibitor for the treatment of various forms of leukemia. The radiosynthesis of [^11^C]ABT-199 has been reported using the “Xantphos-method” and [^11^C]CO [54]. No PET imaging application has been reported as of yet, but future PET studies in animal models are underway. With respect to antitumor agents, Ly573636-sodium (tasisulam) is an antitumor small molecule with a novel mechanism of action that is being evaluated in clinical trials in multiple cancer types, melanoma, lung cancer, and refractory solid tumors [55]. The synthesis of [^11^C]tasisulam using the “Xantphos-method” was reported by van der Wildt et al. and is similarly undergoing evaluation as a PET radiotracer in transgenic mouse models [54]. 

The use of radiolabeled peptides as PET imaging tools for clinical diagnostics has recently attracted considerable attention. Thus, the development of the late-stage radiolabeling of biomolecule-based structures has emerged as a powerful strategy to evaluate novel biopharmaceuticals using PET imaging. Cornilleau et al. reported an interesting method for direct access to [^11^C]CO-labeled biomolecules using Pd(dba)_2_–Xantphos as a catalyst [56]. Two molecules of biological interest were labeled in this study: (i) moxestrol, a steroid ligand with a specificity for the estrogen receptor (ER); and (ii) cyclo-RGD, a cyclopeptide targeting the integrin αvβ3 receptors, which are implicated in different forms of cancer. In a follow-up study, the same group reported the initial evaluation of the [^11^C]moxestrol conjugate as a potential PET radioligand for the in vivo visualization of ERs [57]. Unfortunately, no specific binding was detected in vivo using PET−MRI imaging. A similar late-stage and Pd-mediated carbonylative approach using an isolated methyl–Pd–Xantphos complex was also presented by Andersen et al. to selectively label peptides at the N-terminal or at the internal lysine position with [^11^C]CO [39]. The protocol was later applied to the radiosynthesis of three bioactive peptides, namely [^11^C]lacosamide (Figure 7), [^11^C]acetyl cRGDfK, and [^11^C]SPF-5506-A4. 

## 4. Conclusions

The Pd–Xantphos-mediated ^11^C-carbonylation protocol has allowed laboratories to meet a longstanding unmet need for preparing ^11^C-carbonyl-labeled radiopharmaceuticals at ambient pressure for PET imaging and drug discovery. At our laboratories, the “Xantphos-method” has become the standard method alongside ^11^C-methylation for the ^11^C-labeling of candidate drugs and novel PET radioligands. To date, through the Karolinska Institutet–AstraZeneca partnership, we have labeled more than 30 compounds using this novel method, including two for human PET imaging; furthermore, at the Centre for Addiction and Mental Health in Toronto, we have one new ^11^C-radiopharmaceutical that has been validated for human use using this method (unpublished data). Since its discovery, the “Xantphos-method” has been fully automated for the GMP-compliant production of radiopharmaceuticals, and it has been adapted for “in-loop” reactions and microwave technology. Given the simplicity and efficiency of the method as well as the abundance of carbonyl groups in bioactive drug molecules, we expect that this methodology will be even more widely adapted in future clinical PET radiopharmaceutical research and drug development. 

## Data Availability

Data is contained within the article.
